# Medial Preoptic Regulation of the Ventral Tegmental Area Related to the Control of Sociosexual Behaviors

**DOI:** 10.1523/ENEURO.0283-16.2016

**Published:** 2017-01-09

**Authors:** Onur Iyilikci, Jacques Balthazart, Gregory F. Ball

**Affiliations:** 1 Department of Psychological and Brain Sciences, Johns Hopkins University, Baltimore, Maryland 21218; 2 Department of Psychology, University of Maryland, College Park, College Park, Maryland 20742; 3 GIGA-Neurosciences, University of Liege, 4000 Liege, Belgium

**Keywords:** medial preoptic nucleus, mesolimbic system, social behavior, sociosexual behavior

## Abstract

During sociosexual encounters, different brain mechanisms interact to orchestrate information about the salience of external stimuli along with the current physiological and environmental conditions in order to process these in an optimal manner. One candidate neural system involves the potential interplay between the medial preoptic nucleus (POM) and mesolimbic reward circuitry. We present here evidence indicating that projections originating from the POM play a modulatory role on the mesolimbic reward circuitry related to male sexual behavior in Japanese quail (*Coturnix japonica*). First, we used an asymmetrical inactivation strategy where POM and ventral tegmental area (VTA) were unilaterally inactivated via the GABA_A_ agonist muscimol, either in an ipsilateral or contralateral fashion. Ipsilateral injections of muscimol had negligible effects on both appetitive and consummatory sexual behaviors. In contrast, contralateral injections significantly impaired appetitive sexual behaviors but had no clear effect on consummatory sexual behaviors. Next, we labeled cells projecting from the POM to the VTA by stereotaxic injection into VTA of the retrograde tracer biotinylated dextran amine (BDA). Two weeks later, brains from males who had been allowed to interact freely with a female (15 min) or kept as controls were collected and fixed for double immunohistochemical labeling of BDA and the immediate early gene Fos. More retrogradely labeled BDA cells in POM expressed Fos after sociosexual interactions than in control conditions. Overall, these findings provide novel evidence for the interplay between POM and VTA in the modulation of appetitive but not consummatory sexual behaviors. Schematic representation of the putative role of the projection from the medial POM to the VTA in the regulation of appetitive and consummatory sexual behaviors. Unilateral inactivation of POM and VTA on (1) ipsilateral sides has negligible effects on both aspects of sexual behaviors, whereas (2) contralateral inactivation disrupts appetitive sexual behaviors.

## Visual Abstract

**Figure:**
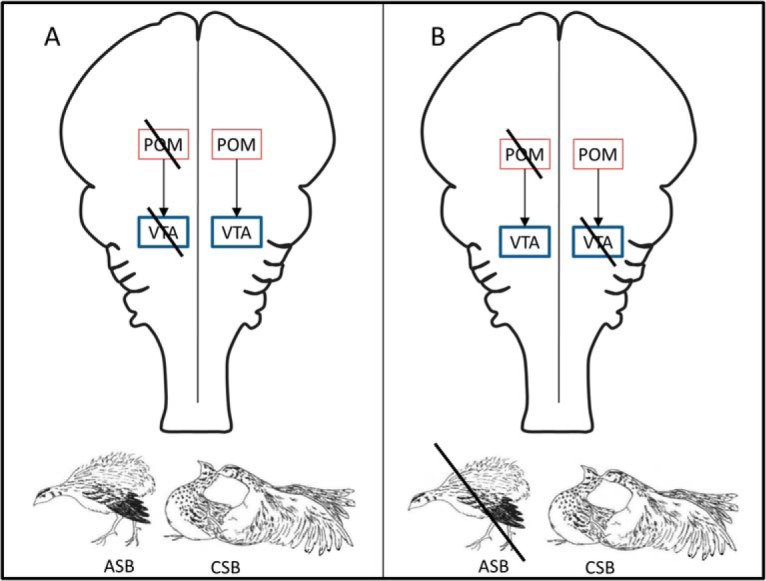

## Significance Statement

Sociosexual interactions have major costs; reproductive behaviors increase energy expenditure as well as risks of predation and disease. The successful coordination of sociosexual interactions with other activities under complex conditions is therefore closely linked to the variation in reproductive success. Optimizing the neural systems mediating this coordination is arguably a key adaptation underlying the evolution of animal populations. The present article provides novel evidence on how projections from the medial preoptic area to ventral tegmental area of the mesolimbic reward circuitry may play a role in mediating the occurrence of appetitive sexual behaviors.

## Introduction

Sociosexual interactions are essential for reproductive success in sexually reproducing species, and, under natural conditions, the occurrence of these interactions comes with intricate challenges and opportunities for animals. Given that these interactions are necessary for individuals’ reproductive fitness, reward mechanisms have evolved to drive males and females to seek out and mate with appropriate conspecifics (O’Connell and Hofmann, 2012). In addition, depending on the specific context in which these social behaviors occur, we would expect animals to vary in their motivational state, depending on the context, and to approach the opportunity for sexual interactions in flexible ways (Ball and Balthazart, 2004; Insel and Fernald, 2004).

Specialized neural mechanisms play diverse roles in regulating responses to these complex external and internal conditions (Insel and Fernald, 2004; Robinson et al., 2008). Recently, a number of behavioral biologists have argued that two major neural systems, the social behavior network and the mesolimbic reward circuitry, interact in the regulation of a wide range of social behaviors (Goodson, 2005; O’Connell and Hofmann, 2011; Goodson and Kingsbury, 2013). A component of the social behavior network, the medial preoptic area (mPOA), is considered to have special importance for the activation of male sexual behavior (SB). The mPOA receives inputs from all sensory modalities and sends projections to motor pathways; moreover, it is responsive to the individual hormonal milieu (Dominguez and Hull, 2005; Will et al., 2014).

The mesolimbic system has also regularly been associated with the rewarding properties of engaging in sexual behavior (Pfaus, 2009). Furthermore, there are a number of findings suggesting that there are indeed significant interactions among the medial preoptic nucleus, preoptic nucleus [POM (the central part of the mPOA)], and the mesolimbic system in avian species. For example, bilateral electrolytic lesions of POM disrupt appetitive sexual behaviors, measured via the learned social proximity response, as well as the male-typical consummatory sexual behavioral responses (Balthazart et al., 1998). Furthermore, the POM is known to be bidirectionally connected to the ventral tegmental area (VTA) in male quail (Balthazart et al., 1994). If the POM is also modulating motivational aspects of sexual behavior, a regulatory interaction of some sort between the POM and the mesolimbic system is thus very probable. The possibility that this sort of interplay occurs in the regulation of male sexual behavior in rats has been noted by other investigators (Will et al., 2014, 2016); however, very few studies have directly tested this hypothesis (Stolzenberg and Numan, 2011; Numan 2014; Will et al., 2014). On the other hand, the mPOA–mesolimbic system interactions are involved in the regulation of maternal behavior in rats. For instance, mPOA lesions disrupt pup-induced immediate early gene immunoreactivity (IEG-ir) associated with maternal behavior in nucleus accumbens (nAc; Stack et al., 2002). Furthermore, contralateral electrolytic lesions of mPOA and VTA disrupt the retrieval behavior of mother rats, whereas ipsilateral lesions do not have an effect on this appetitive aspect of maternal behavior (Numan and Smith, 1984).

These studies indicate that mPOA–VTA connections may have a significant role in the control of appetitive behaviors in a general context. Overall, these latter findings strongly imply that the interplay between mPOA and mesolimbic system might also be critical for the activation of appetitive sexual behavior. To investigate this idea, we used in a first experiment an asymmetric inactivation procedure. We injected muscimol, a GABA_A_ receptor agonist, into the POM and VTA of male quail either ipsilaterally or contralaterally. This procedure results in a temporary inactivation in the nucleus of interest and can suppress neural activity with a short latency in the area of infusion (for review, see Edeline et al., 2002; Allen et al., 2008).

We hypothesized that if the correlated activity of these two nuclei is critical for the expression of appetitive and/or consummatory sexual behaviors, then contralateral inactivation of the two nuclei should disrupt these behaviors. In contrast, ipsilateral inactivation should not have a significant effect, given that both nuclei would still be functioning in a correlated manner in one of the two hemispheres. To investigate further the functional connectivity between POM and VTA, in a second experiment we stereotaxically injected the retrograde neuroanatomical tracer biotinylated dextran amine (BDA; 3 kDa) into VTA. At this molecular weight, BDA retrogradely labels cell bodies that send projections to the injection site (Reiner et al., 2000). By using double-label immunohistochemistry for Fos and BDA, we then examined activation of the cells within POM that project to VTA. We also documented a high number of BDA-positive cells in paraventricular nucleus (PVN) and lateral hypothalamic area (LHA) that projected to VTA; thus, we included these two nuclei in our analysis.

## Materials and Methods

### Subjects

The subjects were 27 experimentally naive adult (∼10 weeks old) male Japanese quail (*Coturnix japonica*). In addition, 10 female Japanese quail were used as sexual stimuli for behavioral tests. All subjects were maintained on a standard 16 h:8 h light/dark cycle at ∼22°C and had food and water available *ad libitum*. All male quail were singly housed in individual cages throughout the experiment, whereas female quail were housed in groups of five. All experimental procedures were in accordance with the Animal Care and Use Committee guidelines of Johns Hopkins University, where the experiments were performed.

### Experiment 1

#### Ipsilateral versus contralateral inactivation of POM and VTA

In the first experiment, 15 male Japanese quail were randomly assigned to one of two experimental conditions: (1) ipsilateral or (2) contralateral cannula placement in POM and VTA and inactivation of these nuclei by muscimol. Before the start of experimental procedures, all male quail were tested to confirm that they were able to exhibit the full suite of stereotypical sexual behaviors. Two permanent unilateral guide cannulae were then stereotaxically implanted and directed at the POM and VTA, either on the ipsilateral or the contralateral side. Following a 1 week recovery period, appetitive and consummatory sexual behaviors of the birds were tested for 4 consecutive days. On days 1 and 3 of behavioral testing, the birds received saline injections, whereas on days 2 and 4 they received muscimol injections 30 min prior to experimental testing. Brains were collected immediately after the last behavioral test was conducted.

#### Surgical procedures and infusions

Prior to surgery, quail were deeply anesthetized with 3–4% isoflurane gas and then placed in a Kopf stereotaxic apparatus. Following the skin incision, a 3.175 mm mounting screw was anchored into the skull near the guide cannula to consolidate cannula attachment. To place the guide cannulae (C315GA/SPC, 26 gauge; Plastics One), two holes in the skull were drilled for POM and VTA according to the following coordinates: VTA was targeted −2.3 mm anteroposterior (AP) from ear bars, ±0.7 mediolateral (ML) from the midline, and −6.0 mm dorsoventral (DV) from dura; POM was targeted +1.8 mm AP from ear bars, ±0.6 mm ML from the midline, and −4 mm DV from dura. The guide cannulae were placed 0.5 mm dorsal to the POM and VTA to minimize any possible damage to the target area. The cannulae were fixed in place with acrylic dental cement and fitted with dummy injectors.

After these surgical procedures, quail were handled daily in the vivarium, and the dummy injectors were removed and reattached so that the birds would habituate to the experimental manipulations. For infusion of muscimol and saline, the dummy injector was replaced by an infusion internal cannula (33 gauge; C315IA/SPC, Plastics One) that was connected via plastic tubing to a Hamilton microsyringe placed in a multiple-syringe pump (KD Scientific). On days 1 and 3, a 0.9% saline solution (0.2 μl) was infused into POM and VTA over a 2 min period. On days 2 and 4, muscimol (100 ng in 0.2 μl; CAS 506044, EMD Millipore) was infused at the same speed. For the last administration, fluorescence-conjugated muscimol (100 ng in 0.2 μl; M-23400, Life Technologies) was used so that we would be able to assess the location and spread of the infusion. All behavioral studies were performed 20–30 min after these infusions. Previous studies (Krupa and Ghazanfar, 1999) have suggested that the effects of muscimol are present as soon as 10 min after administration and persist for several hours.

Fifteen minutes after the last behavioral testing, brains were removed following rapid decapitation and then frozen on dry ice to be stored at −70°C until used. All brains were cut at 50 μm in the coronal plane using a cryostat at −20°C. Subsequently brains were mounted using Vectashield (catalog #H-1400, Vector Laboratories). Photomicrographs of the targeted areas were obtained under a fluorescence microscope (2.5× magnification) for further analysis with ImageJ. Afterward, these images were converted to 8 bit images, and the fractional area of the muscimol spread within each nucleus was calculated. The limits of POM were defined by a rectangular field of 0.50 mm^2^ aligned with lateral edge of the third ventricle that corresponds to plate 13 (interaural, 4.24 mm), and the ventral edge of the anterior commissure and the lateral edge of the third ventricle at a rostrocaudal level, including the largest extension of the anterior commissure that corresponds to plate 14 (interaural, 3.76 mm) of the chicken atlas of Puelles et al. (2007). For VTA, the borders of the nucleus were to be within a rectangular field of 0.45 mm^2^ aligned with the ventral part of the oculomotor nerve that corresponds to plates 30 and 31 (interaural, −0.32 and −0.56 mm, respectively) of the same atlas.

#### Behavioral tests

Behavioral testing took place in an arena (150 × 70 cm) that consisted of three compartments [male holding area (25 cm in length), approach area (100 cm in length), female holding area 25 cm] separated by two transparent sliding panels. First, a female quail was placed at one end of the arena and clearly visible through the transparent Plexiglas panel. Subsequently, a male quail was placed at the opposite end behind the second panel. Thirty seconds later, the transparent panel in front of the male was removed, and the latency of approach to the female quail behind the second Plexiglas glass door was recorded as a measure of appetitive sexual behavior. Two minutes after removal of the first panel, the second panel was removed, and male and female quail were allowed to interact freely during 2 min to test consummatory sexual behavior. The frequency of neck grabs (NGs), mount attempts (MAs), mounts (Ms), and full cloacal contact movements (CCMs) were recorded during this time. The frequency of the strutting display was also recorded during the 2 min when males had no free access to females (for description of these behavior patterns, see Adkins and Alder, 1972; Hutchison, 1978). Frequencies of NGs and Mas. on one hand, and of Ms and CCMs, on the other hand, were very similar, and results concerning only one behavior in each pair will thus be presented to avoid redundancies.

### Experiment 2

#### Retrograde pathway tracing

At the start of the experiment, all subjects (*n* = 12) experienced a 15 min pretest in which males and females interacted freely, which ensured that males were sexually active. Subsequently, BDA (3 kDa) was bilaterally injected into the ventral tegmental area. Following this procedure, male quail were randomly assigned to the following two groups: the SB group and the control group (*n* = 6/group). Fifteen days after the BDA injections, males in the sexual behavior group were placed into the experimental chamber with a sexually active female where they engaged in the species-typical stereotypical appetitive and consummatory sexual behaviors, whereas animals in the control group were placed in the holding cage individually. All animals returned to their home cages after the 15 min manipulation (sexual interactions or holding cage) and remained in their home cages for the next 75 min until their brains were collected.

Birds were anesthetized using isoflurane and placed in a stereotaxic apparatus. BDA (3 kDa, 25 μg in 0.25 μl; catalog #D7135, ThermoFisher Scientific) was stereotaxically injected into the VTA as a retrograde neuroanatomical tracer. The skull was opened above the target brain region and a Hamilton neurosyringe was lowered to the desired coordinates (from ear bars: AP, −2.3 mm; ML, ±0.7 mm; DV, −6.0 mm). Upon reaching the desired dorsal–ventral coordinate, BDA was pressure injected using the Hamilton neurosyringe. The syringe remained in place for 5 min and was then removed slowly. The procedure was then repeated in the VTA of the other brain side. The skin was sutured, and birds recovered under a heat lamp and then were returned to their home cage for 15 d.

#### Fixation and immunohistochemistry

Ninety minutes following onset of the behavioral tests, subjects were decapitated and their brain dissected out of the skull. The brains were placed into acrolein (5% in 0.1 m PBS) for 3 h, washed four times in PBS for 15 min, and cryoprotected in 30% sucrose for 24 h at 4°C. The brains were then frozen on dry ice and stored at −70°C until used. All brains were cut at 40 μm in the sagittal plane using a cryostat at −20°C, and sections were collected in four series.

BDA was visualized in one series of sections using a standard avidin–biotin complex (ABC) horseradish peroxidase staining procedure on free-floating tissue. Sections were transferred to PBS, washed for 5 min three separate times, and then washed in 0.1% sodium borohydride for 30 min. After three additional 5 min PBS washes, tissue was incubated for 30 min in 3% hydrogen peroxide to block endogenous peroxidase activity. After three PBS washes, tissue was incubated for 4 h in ABC. Tissue was washed in PBS and then immersed in sodium acetate for 5 min. Tissue was then exposed to diaminobenzidine (DAB). The reaction was stopped with sodium acetate, and tissue was washed in PBS.

Fos expression was subsequently visualized in the same series of sections by immunohistochemical procedures with the ABC technique. Three rinses with 0.01 m PBS containing 0.1% Triton X-100 were performed between each step. First, sections were incubated for 60 min in 0.3% hydrogen peroxide and in 20% normal donkey serum to remove endogenous peroxidase and decrease nonspecific binding. To minimize the possible cross-reaction between two labels, this step was followed by avidin and biotin blocking (catalog #SP-2001, Vector Laboratories) for 15 min to block possible avidin or biotin binding sites in the tissue. Then sections were incubated in the primary rabbit polyclonal Fos antibody (1:10,000; catalog #sc-52, Santa Cruz Biotechnology) for 48 h. Afterward, sections were incubated for 60 min in donkey anti-rabbit serum. The antibody–antigen complex was localized using the avidin–biotin complex method performed with a Vector Elite Kit (ABC Vectastain Elite PK-6100, Vector Laboratories), and, finally, the peroxidase enzymatic activity was visualized with DAB and intensified with nickel ammonium sulfate and chloride to have a black color. Reactions were terminated by several rinses in PBS. Then sections were mounted on gelatin-coated slides. Slides were dehydrated by exposure to successively increasing concentrations of ethanol and then exposure to xylene and coverslipped using Permount.

These sagittal sections were analyzed under a light microscope at 10× magnification. In all nuclei, quantification was performed bilaterally, and the results presented here are the average of the results from two sides. For POM, the quantification area was a rectangular field (0.2 × 0.7 mm) ventral to the edge of the anterior commissure positioned by the mesh grid eyepiece corresponding to sagittal plates 66–70 (lateral, 0.00–0.96 mm) of the chicken atlas (Puelles et al., 2007). Quantification in the PVN was performed in a rectangular field (0.2 × 0.9 mm) located posterior to the POM at a location that corresponds to sagittal plates 67–70 (lateral, 0.2–0.7 mm) of the chicken atlas (Puelles et al., 2007). For LHA, quantification was performed in a rectangular field (0.9 × 1.2 mm) dorsal to the edge of the optic chiasm in tissue sections that correspond to sagittal plates 71–72 (lateral, 0.96–1.20 mm). Even though all grid placements and counts were made at 10× magnification, the colocalization of the BDA and Fos labeling in the same cells was always confirmed by examination at 40× magnification.

## Results

### Experiment 1

#### Cannula placement

**Figure 1.**
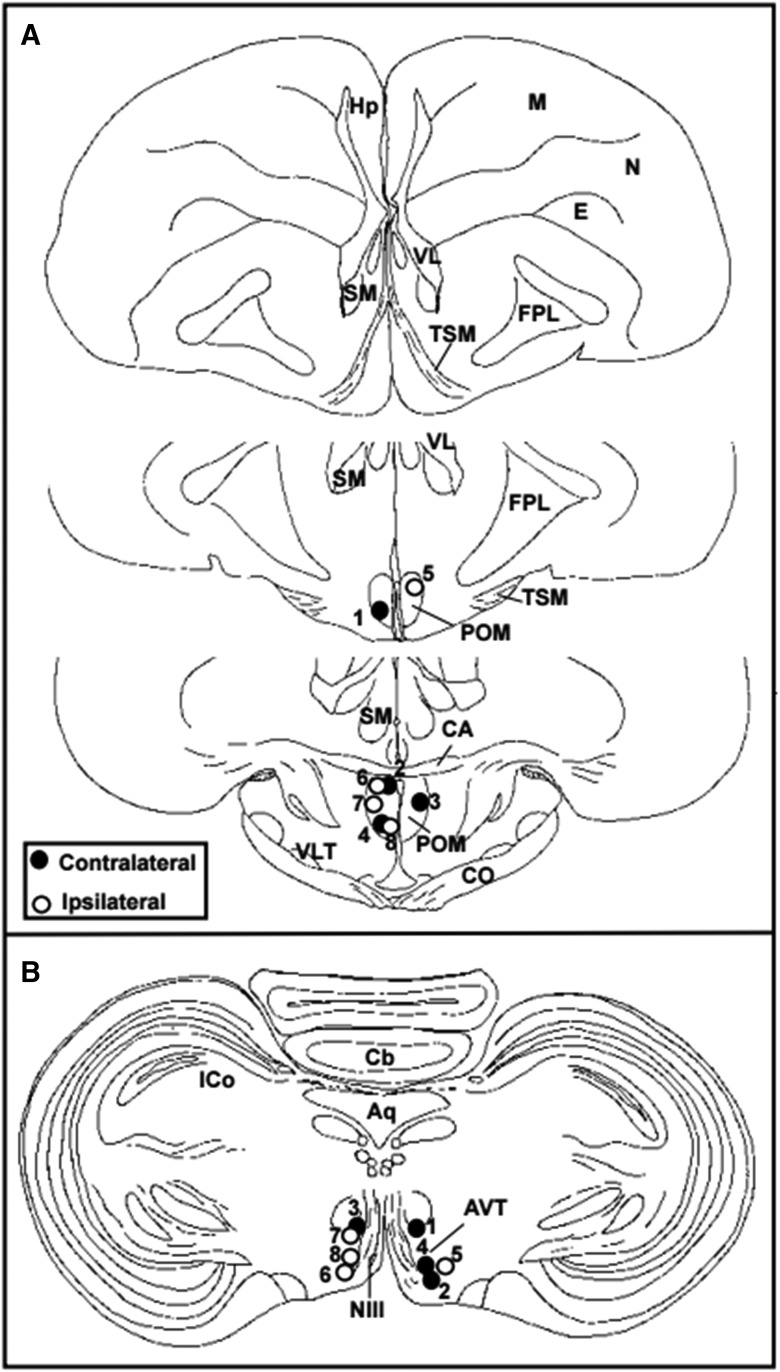
Cannula placements for all intracranial microinjections of muscimol or saline: black dots represent contralateral cannula placements; white dots represent ipsilateral placements. The numbers identify the different subjects and their two cannulae.

**Figure 2.**
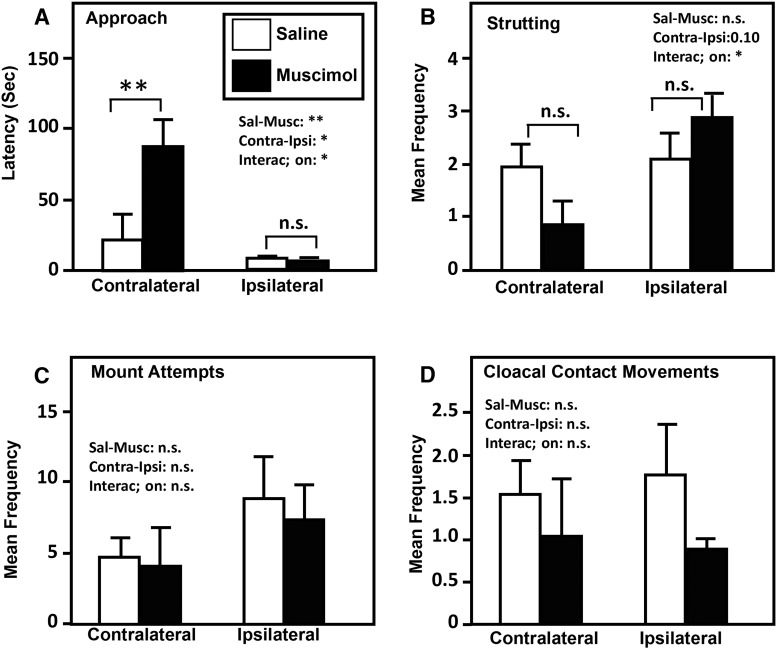
***A–D***, Effects of contralateral or ipsilateral injections of saline or muscimol in POM and VTA on the latency to approach the female (***A***), the strutting frequency (***B***), the frequencies of mount attempts (***C***), and cloacal contact movements (***D***). Data are reported as the mean ± SEM. **p < 0.01, n.s. = *p* > 0.05 by Bonferroni’s *post hoc* tests following a significant interaction in the two-way ANOVA.

Eight of the Japanese quail, four in the contralateral group and four in the ipsilateral group, showed correct cannulae placements in both VTA and POM; other animals (*n* = 7) were excluded from statistical analyses (Figs. 1, 2). Fluorescent muscimol was present in 51.3% (SEM, 2.6%) of the overall POM area in the contralateral group and 54.9% (SEM, 3.2%) in the ipsilateral group. An independent-samples *t* test yielded no significant difference in the area fraction covered by muscimol between two conditions (*t* = 0.909, *p* > 0.05). Muscimol was also present in 61.2% (SEM, 4.8%) of the overall VTA of the contralateral group, and in 64.5% (SEM, 4.5%) of that in the ipsilateral group. No significant difference was present between the two conditions (*t* = 0.498, *p* > 0.05).

#### Behavioral results

Each of the four behavioral dependent variables assessing appetitive (approach latency, strutting frequency during the 2 min when males had visual but not physical access to the female) and consummatory (MAs and CCMs) behavior was analyzed by the same strategy, including first a comparison by a mixed two-way ANOVA of results on T1 and T3, on the one hand (the two tests conducted under saline), and of T2 and T4, on the other hand (the two tests conducted under muscimol), with the two tests being considered as a repeated factor and the two experimental groups (contralateral vs ipsilateral) as independent factor. Since this revealed no significant difference between these pairs of tests (all *p* ≥ 0.196) and no interaction between these test repetitions and the group differences (all *p* ≥ 0.337), the mean results were computed between each pair of tests for each bird and behavioral variable. These data were then analyzed by another series of mixed-design two-way ANOVAs with the type of injections (saline vs muscimol) as the repeated variable and the experimental groups (contralateral vs ipsilateral) as the independent variable.

Analysis of the approach latency identified a significant effect of type of injection (*F*_(1,6)_ = 13.61, *p* = 0.0097, η_p_^2^ = 0.699) and of the experimental group (*F*_(1,6)_ = 8.49, *p* = 0.0268, η_p_^2^ = 0.586). There was also a significant interaction between these factors (*F*_(1,6)_ = 13.61, *p* = 0.0102, η_p_^2^ = 0.694), resulting essentially from the fact that the type of injection had a significant effect in the contralateral group (*t* = 5.248, *p* < 0.01) but not in the ipsilateral group (*t* = 0.029, *p* > 0.05, Bonferroni’s *post hoc* tests).

A similar analysis of the frequencies of struts performed while the male was still separated from the female revealed in contrast no effect of the injection type (*F*_(1,6)_ = 0.2621, *p* = 0.6269) and a nearly significant overall difference between the two experimental groups (*F*_(1,6)_ = 4.636, *p* = 0.0748). There was, however, a significant interaction between these factors (*F*_(1,6)_ = 6.553, *p* = 0.0429). *Post hoc* Bonferroni tests, however, failed to identify an effect of the injection type in both groups even if a tendency was present in the contralateral group (*t* = 2.172, *p* > 0.05).

Finally, analysis of the total frequencies of MA and CCM demonstrated no effect of the injection type (MA: *F*_(1,6)_ = 0.2918, *p* = 0.6085; CCM: *F*_(1,6)_ = 1.599, *p* = 0.2529) or the experimental group (MA: *F*_(1,6)_ = 1.853, *p* = 0.2223; CCM: *F*_(1,6)_ = 0.0193, *p* = 0.8939) and no interaction between the two factors (MA: *F*_(1,6)_ = 0.0495, *p* = 0.8314; CCM: *F*_(1,6)_ = 0.1189, *p* = 0.7420).

### Experiment 2

**Figure 3.**
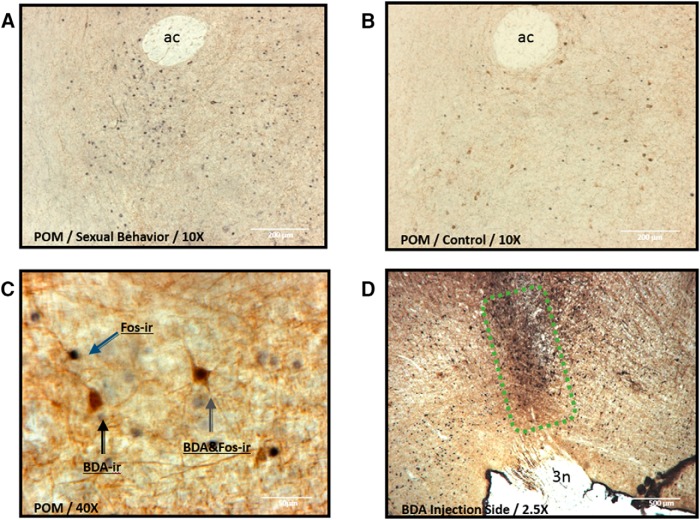
***A***, ***B***, Photomicrographs showing sagittal sections in the quantification area (boxed area) in the POM ventral to anterior commissure (ac) in a male of the SB group (***A***) or of the control (***B***) group. ***C***, Higher-magnification photomicrograph in POM illustrating cells that are Fos immunoreactive (blue arrow), BDA positive (black arrow), or double labeled for BDA and Fos (green arrow). ***D***, Example of an injection site of BDA within VTA dorsal to the oculomotor nerve (3n).

Among the 12 birds that had received a bilateral injection of BDA aimed at VTA, histological verification demonstrated that 8 birds had small injection sites confined to the target structure (Fig. 3D, example). Others had injections that were too large and/or leaked through the cannula track so that they reached the periaqueductal gray and thus could not be used.

Among those eight birds with injections confined to VTA, three had been included into the SB group while the other five were in the control group (assignment was obviously performed before injection sites could be identified). During the behavior test session with a female, all three males in the SB group showed the full copulatory sequence from NG to the CCM. Average frequencies (±SEM) of the sexual behaviors observed during these tests were as follows: NG, 3.7 ± 1.4; MA, 1.7 ± 0.88; M, 3.3 ± 0.88; and CCM, 3 ± 0.58.

This study was designed to test the POM–VTA interplay, and POM was thus the brain area investigated in priority. However, we also detected BDA-positive cells in a relatively large number of brain areas, including the medial septum, habenular nuclei, ventromedial hypothalamus, raphe nucleus, PVN, and LHA. BDA-positive cells were most abundant in PVN and LHA, and we therefore also quantified their presence in these two nuclei as well as their colocalization with Fos in control birds and birds that had been exposed to a female and had expressed sexual behavior.

**Figure 4.**
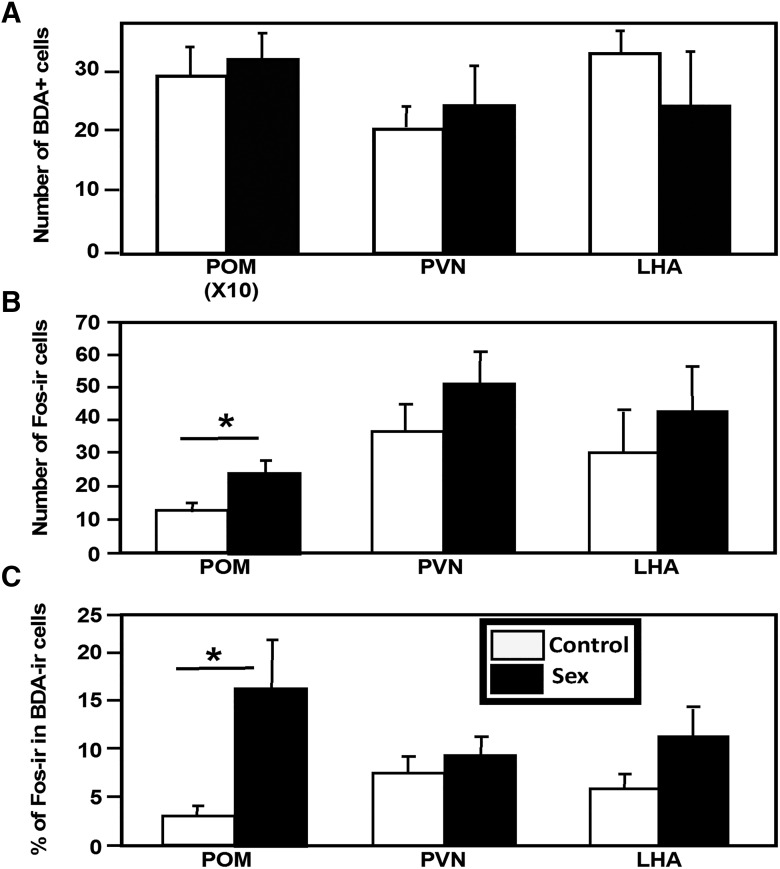
***A***, ***B***, Mean numbers of cells retrogradely labeled by BDA injected into the VTA (***A***) of Fos-ir cells (***B***) and the percentage of the BDA-positive cells that were simultaneously Fos immunoreactive in the POM, PVN, and LHA of the two groups of males that had engaged in sexual behavior (black bars) or had been kept as controls (white bars). Data are reported as the mean ± SEM. *p < 0.05.

As could be anticipated, no difference in the numbers of retrogradely labeled cells was observed between the two groups in all three nuclei (POM: *t* = 0.324, df = 6; *p* = 0.7569; PVN: *t* = 0.5562, df = 6; *p* = 0.5982; LHA: *t* = 1.022, df = 6; *p* = 0.3463; Fig. 4A).

These sections had also been labeled for the Fos protein, and their quantification demonstrated that exposure to the female and the expression of sexual behavior had significantly induced Fos expression in the POM (*t* = 3.787, df = 6, *p* = 0.0091, *d* = 2.61). Similar trends were seen in the other two nuclei, but they were not statistically significant (PVN: *t* = 1.180 df = 6; *p* = 0.2827; LHA: *t* = 0.684, df = 6; *p* = 0.5192; Fig. 4B).

We additionally counted the numbers of double-labeled cells (i.e., neurons that had been retrogradely labeled by BDA injected into VTA and were activated, as evidenced by Fos expression). A small proportion only of BDA-positive cells were immunoreactive for Fos, but this percentage was markedly different between the two experimental groups in the POM (*t* = 3.801, df = 6; *p* = 0.0090, *d* = 2.34) but not in the other two nuclei (PVN: *t* = 0.9077, df = 6; *p* = 0.3990; LHA: *t* = 1.900, df = 6; *p* = 0.1062; Fig. 4C).

## Discussion

Here we demonstrate that ipsilateral inactivation of POM and VTA had negligible effects on both appetitive and consummatory sexual behaviors, whereas contralateral inactivation markedly impaired appetitive sexual behaviors but had no significant effects on consummatory sexual behaviors. In addition, we found elevated levels of immunoreactivity for the immediate early gene Fos in the POM of male quail in response to sexual encounters, while this effect was not observed in the PVN or LHA. Interestingly, this increase in Fos-immunopositive cells was particularly prominent within the BDA-ir cells in POM that project to the VTA, but this effect was not seen in the PVN and LHA. Overall, the results of the current study provide novel evidence for the interplay between POM and VTA in the modulation of appetitive but not consummatory sexual behaviors.

### Ipsilateral versus contralateral inactivation of POM and VTA

Muscimol is a GABA_A_ agonist that can suppress neural activity within a short latency in the area of infusion (Allen et al., 2008). A number of studies have successfully used muscimol-mediated inactivation of the mPOA based on measurements of a variety of behavioral tasks (Hunt et al., 2010; Rusyniak et al., 2011). However, there are conflicting results for the VTA. Some studies reported that a muscimol infusion in VTA facilitates the firing rates of dopaminergic neurons (Xi and Stein, 1998), resulting in an increase in dopamine release in nAc (Xi and Stein, 1998); whereas, other studies using similar manipulations have reported a decrease in dopamine release in nAc (Westerink et al., 1996). These contradictory findings have also been observed at the behavioral level. Some studies have documented that muscimol infusion in the VTA is associated with the augmentation of the behavior of interest, whereas others documented an inhibition (Munro and Kokkinidis, 1997; Doherty and Gratton, 2007; Numan et al., 2009). Numan et al. (2009) suggested that the disruption in the maternal behaviors subsequent to muscimol injections might be due to a hyperactivation of dopaminergic neurons in VTA.

Lesion experiments have previously shown that neural activity in one side of the brain is sufficient to activate male sexual behavior. In gerbils, for example, lesions of the sexually dimorphic area of the preoptic area (SDA) and of the caudal part of the medial bed nucleus of the stria terminalis on the same side of the brain do not inhibit sexual behavior, but lesions on the contralateral sides abolish this behavior (Sayag et al., 1994). The same is true for ipsilateral versus contralateral lesions of the SDA and of the retroruberal field (Finn and Yahr, 1994). We took advantage of this phenomenon to test the notion that it is not only the neural activity in POM and VTA that independently activates behavior in quail, but that it is specifically the interaction between these nuclei that is critical. Indeed, if the separate activity of POM and the VTA was simply synergizing to activate different aspects of sexual behavior, then contralateral muscimol injections in the two nuclei should leave the behavior intact since POM could play its role on one side of the brain, while VTA would play its role on the other side. In this hypothesis, contralateral and ipsilateral inactivation should have a similar behavioral effect. Contrary to this prediction, we observe in experiment 1 that contralateral injections block appetitive behavior, whereas ipsilateral injections do not, thus demonstrating that the activation of behavior requires a functional interaction between the two nuclei in the intact noninjected side.

Note also that this technical approach provides an excellent control for potential nonspecific effects of muscimol since in the two groups of birds (ipsilateral and contralateral injections) all birds received exactly the same amount of the drug in their brain, and it is only when the POM–VTA unit is deactivated on both sides in which the behavior is inhibited.

Although limited, there are some studies indicating a functional significance to mPOA–VTA projections in relation to other motivated behaviors. For instance, in postpartum rats with unilateral lesions of mPOA, exposure to pups induced enhancement of IEG-ir in the contralateral nAc but not on the ipsilateral one where mPOA–VTA projections are disrupted (Stack et al., 2002). Furthermore, contralateral electrolytic lesions of mPOA and VTA disrupted the retrieval behavior of mother rats, whereas ipsilateral lesions did not have an effect on this appetitive maternal behavior (Numan and Smith, 1984). Interestingly, mPOA lesions increased cocaine-induced IEG-ir in nucleus accumbens and conditioned place preference in rats (Tobiansky et al., 2013). Overall, these data indicate that projections from POM to VTA may have a modulatory role in different motivated behaviors.

### IEG and tracing results

In agreement with previous studies, the second experiment revealed an enhanced Fos immunoreactivity in POM subsequent to the expression of sexual behaviors (in quail POM: Meddle et al., 1997, 1999; Charlier et al., 2005; Iyilikci et al., 2014; in mammalian mPOA: Robertson et al., 1991; Heeb and Yahr, 1996; Wersinger and Baum, 1997; Hamson and Watson, 2004; Portillo and Paredes, 2004). In rats, projections from mPOA to VTA have been documented via tract-tracing methods (Simerly and Swanson, 1988), and this projection had also been previously identified in quail (Balthazart et al., 1994; Balthazart and Absil, 1997). The present study confirmed by retrograde tracing from VTA the existence of this projection from POM to VTA in Japanese quail. More interestingly, this study showed that a significantly higher percentage of cells that are projecting to VTA were Fos immunopositive in the males experiencing sexual behavior compared with controls, providing evidence for the functional significance of POM–VTA projections in the regulation of sexual behaviors. Similar trends were observed in the two other nuclei, PVN and LHA, that also demonstrated dense projections to VTA, but the activation as evidenced by Fos expression did not reach statistical significance. Additional work with more subjects and thus more statistical power should determine whether this activation of other nuclei is also physiologically meaningful.

Interestingly, a previous study (Charlier et al., 2005) had also identified, based on Fos induction, an activation of the male quail VTA in birds that had been allowed to copulate with a female, thus confirming its implication in the control of sexual behavior. Furthermore, this activation concerned to a large extent the catecholaminergic cells of the VTA (tyrosine hydroxylase-positive neurons), which is entirely consistent with our contention that the POM–VTA projection modulates the role of the mesolimbic system in the control of appetitive behaviors.

Another important question that remains to be resolved concerns the neurochemical nature of the VTA-projecting POM cells that are activated during sexual interactions: are they steroid sensitive, do they express aromatase, and what is their main neurotransmitter? It would also be important to determine in future experiments whether these cells are activated by the sole expression of appetitive sexual behavior, as potentially suggested by experiment 1 in this article, or whether they express Fos only after the bird has expressed the full copulatory sequence (for such a dichotomy in the quail POM, see Taziaux et al., 2006).

### Medial preoptic area modulates appetitive and consummatory sexual behaviors via separate outputs

In the ethological tradition, many behaviors expressed by animals have been categorized into appetitive and consummatory phases to differentiate goal-directed actions from the stereotypical concluding acts of the goal-directed behaviors. This behavioral dichotomy was first suggested by Charles S. Sherrington (1906), though the terms “appetitive” and “consummatory” were coined by Wallace Craig (Craig, 1918) and have been widely used by ethologists throughout the 20th century (Tinbergen, 1951). This terminology has also been adopted in the study of sexual behavior (Beach, 1956). The usage of these concepts in relation to sexual and related behaviors has been criticized by some researchers (Sachs, 2007), because it can be difficult to establish the exact boundaries between appetitive and consummatory sexual behaviors (Pfaus et al., 2003; Ball and Balthazart, 2008) and the appetitive/consummatory distinction was in the mid-20th century identified with outdated energy models of motivated behaviors (Sachs, 2007). However, it is important to note that hormonal and neuronal manipulations differentially affect behaviors that can be divided into these two categories (Everitt, 1990; Balthazart and Ball, 1998; Ball and Balthazart, 2010). The appetitive/consummatory distinction when used as a way to organize behavioral variation and reduce it to functional categories continues to be useful (Ball and Balthazart 2008).

Early evidence from lesion studies implied that mPOA is a critical structure for consummatory sexual behaviors. Based on additional, more refined experiments, Everitt (1990) argued that mechanisms controlling sexual arousal and copulatory behavior were separate, with mPOA regulating the consummatory sexual behaviors whereas the ventral striatal system would regulate sexual motivation. However, since then, there has been accumulating evidence indicating that POM also plays a role in appetitive sexual behavior in male quail (Balthazart et al., 1998; Tlemçani et al., 2000; Taziaux et al., 2006; Iyilikci et al., 2014; Cornil et al., 2015). For example, *in vivo* microdialysis in the mPOA demonstrated an increase in extracellular dopamine (DA) activity during precopulatory exposure to female conspecifics in male quail (Kleitz-Nelson et al., 2010) and rats (Hull et al., 1995). Furthermore, this precopulatory increase in dopamine was not present in quail that did not subsequently engage in consummatory behaviors. In contrast, the mesolimbic system has been linked predominantly to the regulation of appetitive aspects of these behaviors. It is useful to note that POM–VTA projections have been implicated in the regulations of other neural systems that control motivated behaviors (Stolzenberg and Numan, 2011). The ipsilateral versus contralateral inactivation by muscimol, as used here, provides well controlled evidence indicating a functional role of this projection in the control of appetitive sexual behavior.

The Japanese quail is a photoperiodic species that breeds seasonally during the spring and summer season (Follett and Sharp, 1969; Robinson and Follett, 1982). In male quail, increases in day length cause an increase in the size of the testes (Follett 1976) and in the secretion of steroid hormones that result in the activation of many androgen-regulated traits, such as the cloacal gland in the periphery (Oishi and Konishi, 1983) and the volume of the POM in the brain (Thompson and Adkins-Regan, 1994; Panzica et al., 1996). Therefore, hormonal actions in POM potentially enable the animal to adjust its sexual behavior, possibly including the associated goal-directed (appetitive) behaviors, to a particular physical and developmental condition. POM integrates sensory and hormonal information and regulates these sexual behaviors. Two efferent projections of POM are regulating different aspects of sexual behavior, as follows: (1) previous studies demonstrated that POM projects to the periaqueductal gray, which in turn projects to the brainstem and spinal areas controlling the motor outputs required to engage in stereotypical consummatory sexual behaviors (Ball and Balthazart, 2004, 2010; Wild and Balthazart, 2013); and (2) the present study confirms that the POM also projects to the VTA, and we argue that these projections are involved in goal-directed aspects of sexual behaviors. The dissociations we observed between the contralateral and ipsilateral injection strategies on appetitive and consummatory behaviors, and the increased Fos-ir in the BDA-positive cells of POM are quite consistent with the hypothesis that the POM and VTA are functionally interacting in the regulation of appetitive sexual behaviors. Based on this evidence, we suggest that POM is modulating the mesolimbic system and in this way facilitates the initiation of goal-directed appetitive behaviors via its projections to VTA.

### Conclusion

In the case of male sexual behavior, the costs and benefits of mating behavior are well understood. However, these interactions also have major costs as reproductive behaviors increase energy expenditure as well as risks of predation and disease; thus, coordination of these behaviors in relation to physical and social cues is critical (Adkins-Regan, 2005). In this context, animals should assess the salience of the external stimuli along with the physiological and environmental information and incorporate them to be able to display appropriate sexual behaviors (O’Connell, 2013). The existence of these complex external and internal conditions necessitates an intricate interplay of different brain mechanisms addressing diverse problems faced by an animal to be able to respond in flexible ways. In mammals, dopaminergic projections from VTA to nAc are known to modulate incentive salience in association with a variety of rewarding stimuli (Berridge and Kringelbach, 2008). Potentially, the functional link between POM and VTA facilitates the dopaminergic inputs to nAc, which should lead animals to pay more attention to rewarding stimuli. The present article provides novel evidence on how projections from the mPOA, a key brain site for hormone action, to VTA may play an important role in producing adaptively meaningful responses in the context of sexual behavior and more generally as well.

## Synthesis

Reviewing Editor: Jeffrey Blaustein, University of Massachusetts Decisions are customarily a result of the Reviewing Editor and the peer reviewers coming together and discussing their recommendations until a consensus is reached. When revisions are invited, a fact-based synthesis statement explaining their decision and outlining what is needed to prepare a revision will be listed below. The following reviewer(s) agreed to reveal their identity: Elaine Hull, James Pfaus.

The reviewers were very enthusiastic about your manuscript, but they have noted quite a few grammatical and typographical errors. Please tend to all of these throughout the manuscript. In addition, although eNeuro does not require statements of effect size, we suggest that you include them as discussed in a recent commentary in the journal (http://eneuro.org/content/3/4/ENEURO.0030-16.2016). Reviewer 2 suggests that much of the discussion from the subhead on line 411 to the conclusion on line 485 could be condensed.

Finally, the visual abstract would benefit from including indication that strutting is an appetitive behavior. I have included the full reviews to facilitate your identifying many, but not all, of the errors.

Reviewer 1:

This manuscript reports that contralateral, but not ipsilateral, inhibition of the medial preoptic nucleus (POM) and ventral tegmental area (VTA) impaired appetitive, but not consummatory, sexual behavior of male quail. The authors summarize previous data showing that the POM is important for consummatory sexual behavior in quail and in rats and also contributes to appetitive reproductive behavior. Apparently, unilateral activation of the POM is sufficient to elicit consummatory behavior via non-VTA projections, but it needs its projections to the VTA, and therefore activation of the mesolimbic dopamine tract, to elicit appetitive behavior. Scientifically, the manuscript is interesting and contributes to the appetitive vs. consummatory distinction. There are many grammatical errors, and I do not have time to correct all of them. I will list some:

Line 29: Add “of” (occurrence of sexual behaviors)

Line 82: Add a comma after “effect.”

Line 88: Add “a” (documented a high number); remove the comma after PVN.

Line 89: Add a semicolon after“VTA.”

Line 106: “permanent unilateral guide cannulae”

Lines 109 & 110: “days one and three” “days two and four” Also, add a comma after “saline injections.”

Line 120: Change the comma after “dura” to a semicolon.

Lines 122 & 123: change “cannula was” to “cannulae were.”

Line 126: Add “were.” (”injectors were removed”)

Line 131: Don't capitalize “Muscimol.”

Line 142: Change “are” to “were.”

Line 149: Change “was” to “were.”

Line 175: “sexually active female”

Line 211: Add a comma after “two labels.”

Line 217: Don't capitalize “Nickel.”

Lines 221-222: Reword: “For all nuclei, quantification was performed on both sides of the brain, and....”

Line 238: Change the comma to a semicolon.

Lines 252-254: Be consistent with capitalization of the behavioral labels, either first letters of all words capitalized or all lower case. Also, remove the middle “c” from “Clocacal” (Cloacal)

Line 256 & 257: Add “T” (T1 and T3). Don't capitalize “Muscimol.”

Line 267: Remove one of the double parentheses. “((F1.6....)”

There are numerous additional places where commas are needed to separate clauses, but I don't have time to list them all.

Line 315: Add “of.” (numbers of double-labeled....)

Lines 345-346: The authors cite two studies showing inhibition of behaviors by muscimol in the VTA, but cite no studies showing augmentation. Also, add a comma after “behavior of interest.”

Line 354: Change “contral lateral” to “contralateral.”

Line 357: “Indeed, if the separate activity of POM....” (Add a comma and “of.”)

Line 368: Change “VAT” to “VTA.”

Line 441: Add “of” (”appetitive aspects of these behaviors.”

Line 475: Don't capitalize “Reviewed.”

Reviewer 2:

This study shows a functional relationship between the POA and VTA in the control of appetitive sexual behavior in male quail. Given the roles shown previously for both regions separately (in rats, quail, and voles), the evidence provided here demonstrates unequivocally that their interaction is critical for behavioral indices of sexual desire. It is an excellent paper, clearly written, and with very sound integrative methodology. My only concerns have to do with some minor typos (e.g., line 368, “POM-VAT” that should read “POM-VTA”), the need to include estimates of effect size with their statistical analyses, and my impression that the discussion is a bit overlong. Much of the discussion from the subhead on line 411 to the conclusion on line 485 could be condensed (e.g., the two outputs from POM), or left out entirely (the discussion of ventral pallidum which was not examined in the present experiment).
